# Hospitals alike, hospitals different: spatial clustering of inequality around Boston’s hospitals

**DOI:** 10.3389/fsoc.2026.1736351

**Published:** 2026-06-10

**Authors:** Eralp Kaan Karduz

**Affiliations:** Department of Sociology, University of Massachusetts Boston, Boston, MA, United States

**Keywords:** healthcare access, hospital closures, hospitals, racism, segregation, spatial analysis, urban health

## Abstract

This brief research report investigates how racial and socioeconomic segregation shape spatial inequalities in health outcomes and healthcare access across Boston, Massachusetts. It examines the spatial clustering of race, poverty, and poor health, and analyzes how these patterns align with the locations of major hospitals in the city. We integrated data from the 2018–2022 American Community Survey, CDC PLACES, and the Massachusetts Center for Health Information and Analysis (CHIA) for all non-specialized acute care hospitals in Boston. Spatial autocorrelation was assessed using Global Moran’s *I* statistics and Bivariate Local Indicators of Spatial Association (BiLISA) maps to identify co-clustering of non-White population, poverty, and poor health at the census tract level. All three measures showed statistically significant positive spatial autocorrelation (*p* < 0.001), confirming moderate-strong spatial clustering across Boston. Bivariate maps show racial composition, poverty, and poor health outcomes to have significant spatial clustering around hospitals. Historical segregation patterns still exist to this day and shape how people receive healthcare in the city. Patterns around recently closed Carney Hospital further underscored the spatial burden of care redistribution. Safety-net hospitals disproportionately serve neighborhoods with the greatest health and economic vulnerabilities while wealthier hospitals are embedded in healthier areas. These findings highlight the need for interhospital collaboration, equitable resource distribution, and policy interventions that explicitly address the spatial legacy of structural racism in healthcare delivery in American cities.

## 1 Introduction

Racial segregation has been documented to be the basis of the formation of socioeconomically disadvantaged communities in American cities (Massey, 1990). Historically, Boston, as one of the largest cities on the East Coast, has been a focal point for studies of racial and socioeconomic segregation from many perspectives including education (Kantrowitz, 1979; Ross et al., 1966) and housing (Schafer, 1979; Guy, 2000). Despite what seems to be progress in reducing black-white segregation, the growing ethnic diversity in U.S. cities shows that minority groups continue to experience persistent residential segregation (De la Roca et al., 2014). Recent studies argue that the persistence of segregation cannot be fully understood without attending to contemporary urban changes, such as growing ethnic diversity, shifts in socioeconomic structures, and widespread urban redevelopment (Hwang and McDaniel, 2022). In particular, the interaction of race and class, rather than either one of them alone, has become a defining feature of urban spatial inequality.

This study addresses two key gaps in the existing literature: first, by providing a geospatial analysis of how racial and socioeconomic segregation intersect with health outcomes in Boston; and second, by contextualizing the spatial relationship between these clusters and healthcare utilization. It asks: How are racial composition, poor health, and poverty spatially clustered across Boston, and how is this clustering locally patterned around hospitals?

## 2 Literature review

### 2.1 Conceptual framework

Research on social determinants of health and the socio-spatial distribution of health outcomes has grown over the past two decades, particularly at the intersection of neighborhoods and health (Diez Roux and Mair, 2010). This reflects a broader shift within public health scholarship, which increasingly integrates more sophisticated theoretical frameworks linking health outcomes to environmental and social context, moving beyond biologically reductionist approaches (Merz et al., 2023). Ecosocial Theory builds on this shift, proposing that people embody their lived experiences in social and ecological contexts through pathways such as social trauma and differential access to resources (Krieger, 2012; Krieger, 2001); bodies thus serve as physical records of lifelong exposures that are biologically inscribed over time and produce observed patterns of population health (Krieger, 2014). Racial segregation is one of the most consequential of these pathways, considered “a fundamental cause of racial differences in health” (Williams and Collins, 2001), and the structural inequalities it produces play a central role in differential health risks across communities (Kramer and Hogue, 2009). The three variables clustered in this study—racial composition, poverty, and poor health (chronic disease prevalence)—operationalize this concept of embodiment at the neighborhood scale.

### 2.2 Empirical context

Health disparities are sustained through segregation, and access to healthcare has been one of the most prominent areas of research in spatial urban disparity studies (Diez Roux and Mair, 2010; Malaker and Meng, 2024). Geographic location plays a significant role in how one receives healthcare services, especially for minority populations that reside in areas where healthcare access and quality are diminished (Baicker et al., 2005). Structural racism through gentrification has also been linked with health outcomes across the US, through both direct mechanisms, such as displacement, and indirect ones, like community belonging (Versey, 2023).

Hospitals are geographically fixed in place, even as the spatial and social characteristics of surrounding neighborhoods constantly change. The contemporary spatial pattern of hospital care is also shaped by an explicit history of segregationist policy. The Hospital Survey and Construction Act of 1946 permitted federally funded hospitals to remain racially segregated, and significant gaps in equitable access persisted long after the Civil Rights Act of 1964 and the Fair Housing Act of 1968 formally prohibited such practices (Benjamins et al., 2021). Still today, hospital segregation often mirrors residential segregation; Black and White patients frequently receive care at different facilities (Himmelstein et al., 2023; Kung et al., 2025; Lin et al., 2023). Black patients are disproportionately served by lower-performing hospitals compared to White patients (Chandra et al., 2024). In racially segregated neighborhoods, Black patients are disproportionately likely to undergo surgery in low-quality hospitals, whereas in more integrated areas, Black and White patients are equally likely to receive care at lower-quality facilities (Dimick et al., 2013). Hospitals serving a large proportion of Black patients are also systematically under-resourced compared to other hospitals (Himmelstein et al., 2023).

Urban hospitals primarily interact with many of their low-income and uninsured neighbors through emergency departments (EDs) (Skinner et al., 2023). These facilities are frequently overcrowded and under-resourced (Savioli et al., 2022), yet marginalized populations who are underrepresented in preventive care often rely on emergency services even for chronic and life-sustaining treatments (Roberts, 2012). While these patterns have been documented at the national level, they take on more historically grounded forms in individual cities (McKee, 2016).

### 2.3 Boston as a case

In Boston, the story of racial segregation and disinvestment traces back to the 19th century, when ethnic groups such as the Irish and Italians practiced voluntary segregation, but Black residents faced more systemic racial exclusion (Kantrowitz, 1979). By the end of the 20th century, three decades after the federal Fair Housing Act, the Boston metropolitan housing market remained sharply segregated and Black and Latino homebuyers were largely absent from the areas where real estate values were rising fastest (Guy, 2000). Even after redlining was officially outlawed, banks continued to approve loans at significantly lower rates in these neighborhoods and continued racially motivated urban disinvestment (Taggart and Smith, 1981). Some parts of the city followed a different trajectory: Jamaica Plain, for example, was produced as a White space through settler colonial place-making and well documented gentrification (Hermann et al., 2019; Montalva Barba, 2024).

Today, the health consequences of redlining remain visible as neighborhoods experience higher rates of food insecurity, reduced access to grocery stores, and an increased prevalence of chronic diseases such as hypertension, diabetes, and obesity (Mehrtash, 2025). In the area, studies on diabetes, COVID-19, and premature mortality have consistently shown that economically disadvantaged neighborhoods or those with higher concentrations of minority populations, experience significantly worse health outcomes (Piccolo et al., 2015; Samuels-Kalow et al., 2021; Chen et al., 2006). Despite these persistent inequities, Boston has one of the lowest differences between Black and White life expectancy among largest US cities (Benjamins et al., 2021). At the same time, according to the Lown Institute’s (Lown Institute, 2024) hospital inclusivity ranking—based on how well a hospital’s patient population reflects the demographics of its surrounding community in terms of race, income, and education levels—Boston stands out for hosting both one of the most inclusive hospitals in the country (Boston Medical Center, ranked 2 out of 2,616) and one of the least inclusive (Brigham and Women’s Hospital, ranked 2,238).

Hospital closures also add another layer to this pattern as they are directly linked to the percentage of Black residents in the neighborhood (Smith, 1999). Between 1970 and 2010, 45% of hospitals open closed and 60% of these closures happened in predominantly Black neighborhoods (Yearby, 2018). In Massachusetts, the number of acute care hospitals went down to 70 in 2025 [MassGIS (Bureau of Geographic Information), 2025] from 110 in 1980 (Sager and Socolar, 2002). Hospital closures, particularly in urban areas, force patients to travel farther for medical care, increasing wait times for emergency services and specialty treatments, particularly for those with chronic conditions who rely on consistent care (Romero et al., 2012). In addition to loss of access to care, hospital closures also represent a loss of local employment and economic capacity for the community (Alexander and Richards, 2023; Egede et al., 2023). While some closures result in efficiency gains for remaining hospitals, the overall impact on the healthcare system is not as straightforward, as many patients experience increased costs and difficulty securing care (Mullens et al., 2023). The recent closure of Steward Carney Hospital in Boston (Steward Corporate, 2024) provides an example of these dynamics and raises critical questions about the redistribution of patient load from its former catchment area. This context highlights the urgent need for the analysis this study undertakes.

## 3 Method

### 3.1 Data

Center for Health Information and Analysis (2024) data is used for hospital profiles including financial performance data, utilization data, and data for communities with the most number of discharges from each hospital. This analysis includes all non-specialized acute care hospitals in Boston operational as of September 2025: Brigham and Women’s Hospital, Massachusetts General Hospital, Beth Israel Deaconess Medical Center (hereinafter “Beth Israel”), Tufts Medical Center, Boston Medical Center (hereinafter “BMC”), St. Elizabeth’s Medical Center (subsequently renamed BMC Brighton following its acquisition by BMC Health System), and Brigham and Women’s Faulkner Hospital (hereinafter “Faulkner”) (Boston Public Health Commission, 2024). Carney Hospital, which closed in the summer of 2024, is also included in the discussion and Supplementary material. Non-specialized acute care hospitals were selected because they provide majority medical, surgical, and emergency services to the general adult population, and therefore play a central role in the city’s overall healthcare access and outcomes. Specialized hospitals, such as Boston Children’s Hospital, Dana-Farber Cancer Institute, and Massachusetts Eye and Ear Infirmary, were excluded because they primarily serve specific patient groups and are not representative of access to care for the broader population.

Secondary socioeconomic and health data across census tracts within Boston were used in the analysis. Data on race and poverty were drawn from the 2018–2022 American Community Survey 5-year estimates. ACS is an annual survey conducted by US Census Bureau (2023) to gather data on socioeconomics and demographic characteristics of the U.S. population. The 5-year estimates aggregate data collected over a five-year interval to provide reliable estimates for smaller geographic units such as census tracts. Finally, health outcome data are obtained from the CDC’s PLACES dataset (formerly known as the 500 Cities Project) (National Center for Health Statistics, 2023), which provides census tract–level estimates of health outcomes, risk behaviors, and social determinants of health.

### 3.2 Measures

*Non-White population* is measured by the percentage of the non-White population in each census tract. *Poverty* is defined as the percentage of the population living below 150% of the federal poverty line in each census tract. This threshold was selected to more accurately reflect financial strain in Boston, where high living costs render the federal poverty line an underestimation of local economic hardship. *Physical health outcomes* from the CDC’s PLACES dataset were consolidated into a single variable to measure overall poor health. This was done by conducting principal component analysis (PCA) using the rates of six major chronic conditions: cancer, coronary heart disease, diagnosed diabetes (Type I and Type II), high blood pressure, high cholesterol, and stroke, all among adults over the age of 18. These conditions were selected based on data availability, their contribution to overall morbidity (National Center for Health Statistics, 2025), and their persistent effect throughout the life course (Lynch and Smith, 2005) All six conditions loaded strongly and positively on the first principal component (loadings ≈ 0.80–0.89) supporting interpretation of the index as a neighborhood-level measure of overall chronic disease concentration (see Supplementary Table 1 for factor loadings).

### 3.3 Analytic approach

Univariate Global Moran’s *I* statistics were computed to assess spatial correlations among variables across Boston. Global Moran’s *I* provides a summary measure of whether similar values of a variable are spatially clustered or randomly distributed (Moran, 1950). This is to confirm whether spatial autocorrelation exists at the citywide scale. Global Moran’s *I* values were first estimated using the Spatial Dependence package in R (Bivand and Wong, 2018; R Core Team, 2024). While this statistic has been widely used for summarizing overall spatial autocorrelation (Bivand and Wong, 2018), it might obscure local patterns that are not aligned with the global spatial trend (Anselin, 1995).

Bivariate Local Indicators of Spatial Association (BiLISA) maps were used to examine local spatial associations. BiLISA maps examine the value of one variable at a location and the average of neighboring values for the second variable and allow for the identification of local clusters and spatial outliers. High-High (HH) clusters are census tracts where high values of a variable are surrounded by high values of spatial lag of the other variable. Similarly, Low-Low clusters are where the low values of the first variable are surrounded by low values of the spatial lag. High-low and Low-High clusters represent census tracts that differ significantly from their surrounding areas—i.e. spatial outliers (Anselin, 2024). BiLISA maps are constructed for three pairs of variables: non-White Population–Poverty, non-White Population–Poor Health Index, and Poverty–Poor Health Index, using spdep. Both global Moran’s I and BiLISA maps are descriptive, exploratory tools and are not intended as evidence of causal spatial association.

All spatial analyses used first-order Queen contiguity matrix for both global Moran’s I and BiLISA, whereby two census tracts were considered neighbors if they shared a common edge. For areal units, such as census tracts, contiguity-based weights better represent the spatial structure (Bivand and Wong, 2018; Stakhovych and Bijmolt, 2009; Gibbons et al., 2020). Table 1 also reports Global Moran’s I under alternative standard weight constructions. Because the substantive cluster patterns (especially large clusters) are similar across different spatial weights (cluster agreement ranged from 82.5 to 93.7%), we report queen contiguity BiLISA results in the main text for consistency (Anselin, 1995) and parsimony (LeSage and Pace, 2014) (see Supplementary Table 3).

**Table 1:** Global Moran’s *I* for selected variables across Boston, 2022.

Year	Measure	Global Moran’s *I*				Spatial clustering
		Inverse distance weights (IDW), *p* = 1	Rook contiguity (1st order)	Queen contiguity (1st order)	k-nearest neighbors, kNN (Diez Roux and Mair, 2010)	
2022	Poor health index	0.52***	0.56***	0.57***	0.50***	Strong
	Non-white population %	0.58***	0.60***	0.58***	0.56***	Strong
	Poverty %	0.25***	0.28***	0.27***	0.22***	Moderate
2017^1^	Poor health index	0.53***	0.53***	0.53***	0.51***	Strong
	Non-white population %	0.69***	0.69***	0.72***	0.67***	Strong
	Poverty %	0.32***	0.32***	0.31***	0.32***	Moderate

*p < 0.05, **p < 0.01, ***p < 0.001.

^1^2017 Data included for sensitivity analysis.

Hospital proximity has been shown to influence whether, when, and how individuals seek care (Ingram et al., 1978). However, hospital catchment areas are not mutually exclusive and are poorly approximated by assigning each resident to a single nearest facility (Delamater et al., 2019). Accordingly, this study employs a floating catchment approach for hospital service areas to summarize provider and population availability within a reasonable access threshold (Yang et al., 2006; Luo, 2004). Tract-level potential access was calculated using a two-step floating catchment area framework in which a 15-min driving time threshold defined facility and population catchments. Hospital bed supply was first converted to a local supply-to-demand ratio within each hospital’s catchment and then summed across all hospitals within each tract’s catchment to yield beds per 1,000 residents, allowing overlapping catchments, mapped with and without Carney Hospital (Supplementary Figure 1) (Delamater, 2013).

## 4 Results

To address the first part of the research question, global spatial autocorrelation of racial composition, poor health, and poverty across Boston is examined. Table 1 shows the results of univariate Global Moran’s *I* statistics for each measure. All three variables show statistically significant positive spatial autocorrelation in 2022. The Poor Health Index had a Moran’s *I* of 0.57 in 2022 (and *I* = 0.53 in 2017, *p* < 0.001), indicating strong spatial clustering, census tracts with poor health outcomes tend to be located near others with similar conditions across Boston. Similarly, non-White population also shows high spatial autocorrelation (*I* = 0.58 and a higher *I* = 0.72 in 2017, *p* < 0.001). Finally, there is a slightly less strong spatial pattern for poverty rates across Boston with *I* = 0.27 in 2022 and *I* = 0.31 in 2017 (*p* < 0.001).

**Figure 1:**
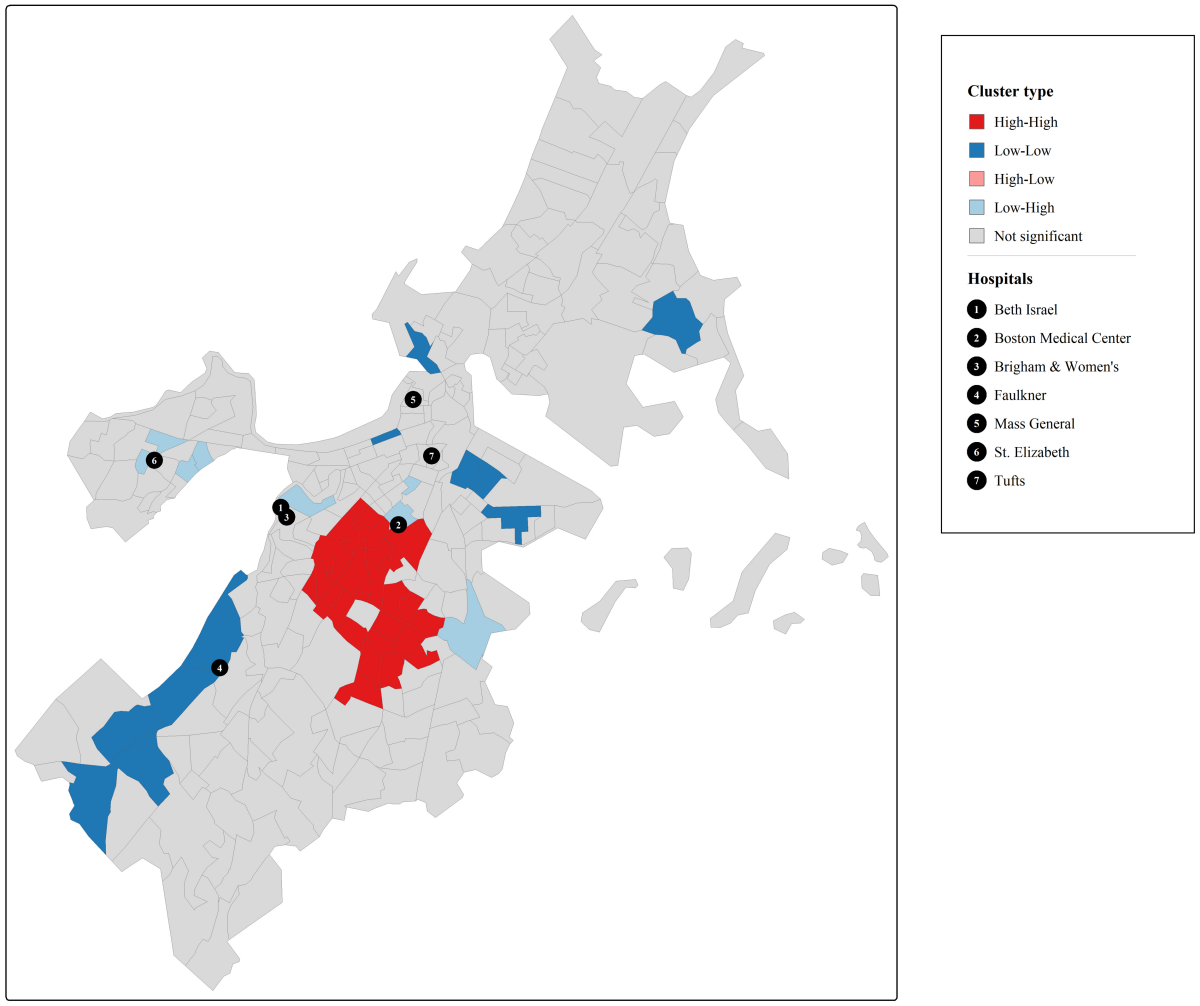
BiLISA map, non-white population % and poverty % across Boston census tracts, 2022.

**Figure 2:**
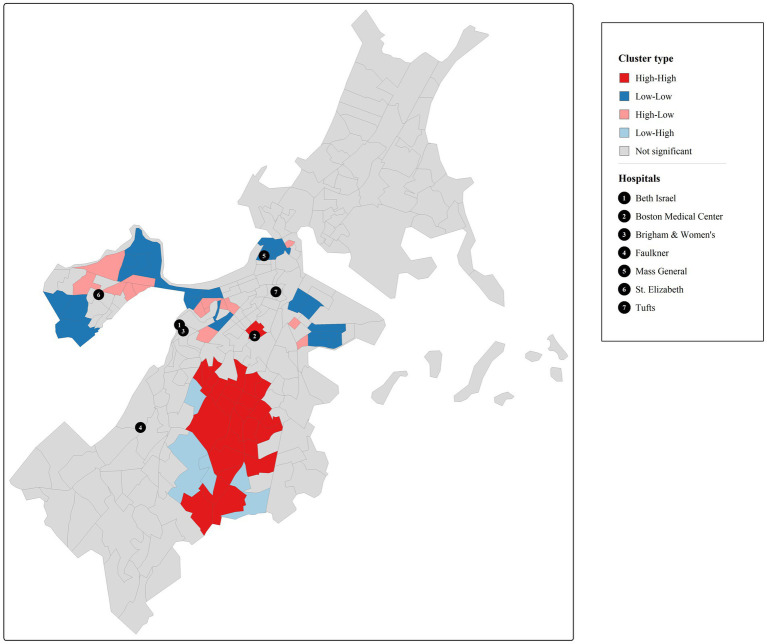
BiLISA map, poverty % and poor health index across Boston census tracts, 2022.

**Figure 3:**
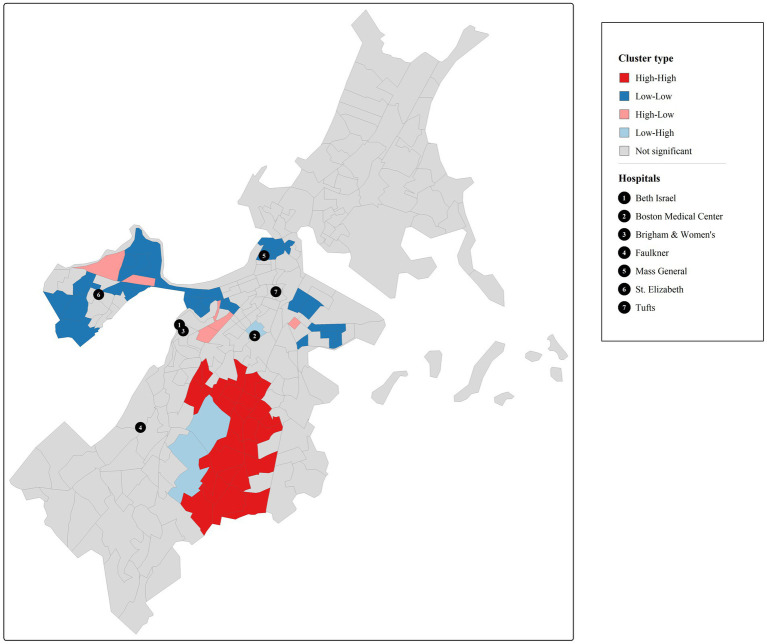
BiLISA map, non-white population % and poor health index across Boston census tracts, 2022.

To address the second part of the research question- how this clustering is patterned around hospitals- hospital locations are overlaid onto the bivariate local Moran’s *I* maps. Figure 1 shows the BiLISA map for poverty rate and non-White population. There is a larger cluster of HHs in Roxbury and Dorchester and LL clusters in West Roxbury, JP, and East Boston. Figure 2 shows the bivariate BiLISA map for poverty rate and poor health. In the South of the map, around Dorchester, Mattapan, Roxbury, and Hyde Park neighborhoods, there is a large cluster of HHs and a couple of LHs on the western edge of the cluster towards Jamaica Plain (JP). This cluster is largely around BMC and Faulkner. Then around St. Elizabeth, Brigham and Women, and Beth Israel, there are HL outliers surrounded by LL tracts. In the South Boston neighborhood, there are also LL clusters. Figure 3 shows the bivariate BiLISA map for the non-White population and poor health. The significant areas are similar to Figure 2, with more LH outliers towards Hyde Park and HL outliers around Allston and Brighton. Across all three figures, there are also LL clusters around Allston, Brighton, Fenway-Kenmore, Back Bay, and South Boston neighborhoods.

## 5 Discussion

The magnitude of the univariate Global Moran’s *I* statistics indicates that these outcomes are spatially autocorrelated to different degrees. The Poor Health Index (*I* = 0.57) shows strong spatial association, suggesting that poor health is organized into broader neighborhood-scale zones rather than isolated tracts. In line with the previous literature (Gibbons et al., 2020), our results quantify the strength of the neighborhood structuring at a fine spatial resolution and in a recent time point. Similarly, non-White population shows strong spatial association (*I* = 0.58), consistent with what is expected from historically segregated cities. This is also consistent with an Ecosocial reading of segregation as a pathway through which structural inequality becomes biologically inscribed at the neighborhood scale. Global Moran’s *I* for the non-White population and Poor Health Index are both stronger than poverty (*I* = 0.25). Poverty, specifically, may show weaker global clustering because of the gentrification in low-income tracts (Hermann et al., 2019), “scattered site” public housing (Boston Housing Authority, n.d.). These global patterns and more specific outcomes (i.e., life expectancy, segregation) have also been recognized by the city (Boston Public Health Commission, 2025) and are more apparent when looking at bivariate clustering.

Together, the BiLISA maps reveal three distinct hospital contexts: (Massey, 1990) hospitals in predominantly HH clusters across race, poverty, and poor health (e.g., BMC and Faulkner) (Kantrowitz, 1979) hospitals in predominantly LL clusters (e.g., Mass General, Tufts), and (Ross et al., 1966) hospitals situated in mixed or transitional zones characterized by HL/LH outliers (e.g., Beth Israel, St. Elizabeth, and Brigham and Women). The remainder of this section will consist of two comparisons: “hospitals alike,” hospitals in similar neighborhood clusters (i.e., comparable LL or HH contexts), and “hospitals different,” hospitals in different clusters of inequity across the city. From a policy perspective, hospitals in wealthier and healthier areas could leverage and mobilize their resources to meet the needs of patients and other hospitals across the city, especially at times of hospital closures and federal funding cuts.

### 5.1 Hospitals alike

Beth Israel and Brigham and Women are co-located in the Longwood Medical Area (LMA), a major medical hub that employs more than 73 thousand people and treats 3.8 million patients annually. The LMA received nearly $1.4 billion in NIH funding in 2025, which is more than the funding received by 43 individual states (City of Boston Planning Department Research Division, 2026). St. Elizabeth, located further west, shares a similar non-HH clustering pattern with Beth Israel and Brigham and Women. Their surrounding tracts are predominantly non-significant, LL, or outliers (LH in Figure 1 and HL in Figures 2, 3). Their immediate neighborhoods have lower-than-average non-White populations, lower poverty rates, and better health outcomes relative to the city as a whole, and their service areas appear well-resourced in terms of hospital bed accessibility (see Supplementary Figure 1).

The HL outliers in Figures 2, 3 offer further nuance within this similar spatial context for Beth Israel, Brigham and Women, and St. Elizabeth. In Figure 2, scattered HL tracts are present around Allston, Brighton, and Fenway-Kenmore. Parts of these neighborhoods have historically been working-class, student-heavy areas that have undergone gentrification, with economically vulnerable populations (Hosman, 2018). In Figure 3, there are also a few HL outliers, similar to those in Figure 2, in the area around the LMA and St. Elizabeth. These outliers are useful for pinpointing smaller areas where health outcomes might be different than what poverty or racial composition alone would predict. The presence of these outliers in hospital-dense areas is notable, particularly given elevated demographic risk. While proximity to healthcare resources is one plausible contributing factor, other tract-level characteristics not captured in this analysis, such as insurance coverage or access to social services, may also explain why better health outcomes are clustered in these tracts.

Proximity to hospitals and favorable neighborhood demographics do not guarantee equitable access to care. While these hospitals share a similar spatial context, St. Elizabeth diverges sharply from Beth Israel and Brigham and Women in institutional capacity and the populations they serve. Beth Israel and Brigham and Women are both Harvard Medical School teaching hospitals that have long operated as nonprofit academic medical centers within the LMA’s deep concentration of resources and research funding. St. Elizabeth, by contrast, was previously a for-profit teaching hospital and only recently transitioned to nonprofit status under the BMC Health System after the bankruptcy of its previous owners, Steward Health Care. Moreover, St. Elizabeth also serves different demographics compared to Beth Israel and Brigham and Women. St. Elizabeth accounts for 42% of all discharges among Brighton residents and serves a population with greater need for subsidized healthcare. Meanwhile, Beth Israel and Brigham and Women, for example, together account for approximately 67% of discharges among Brookline residents, a predominantly white and wealthy town directly adjacent to LMA (Center for Health Information and Analysis, 2024). Financially, St. Elizabeth generates less than a quarter of Beth Israel’s revenue and almost one-tenth of Brigham and Women’s revenue, operating with fewer staff and beds than both (see Supplementary Table 2). Ultimately, while the three hospitals appear geospatially alike, St. Elizabeth operates in an entirely different reality in some important aspects. From an equity standpoint, addressing this profound resource asymmetry between well-capitalized academic medical centers and financially strained community hospitals serving higher-need populations is critical to improving outcomes across their overlapping service areas.

### 5.2 Hospitals different

The “Hospitals Different” contrast is strongest between hospitals embedded in the south/central HH corridor and those surrounded by LL or non-significant tracts. In Figures 2, 3, Roxbury–Dorchester–Mattapan form a contiguous HH cluster (high poverty paired with poor health; high non-White population paired with poor health), and Figure 1 shows the same geographic clustering as HH for non-White population and poverty. BMC is positioned near this HH cluster, which helps explain why it might carry a disproportionate safety-net burden and why these clusters have a higher reliance on BMC’s emergency department. In contrast, Beth Israel is located in a service geography that is primarily LL or non-significant on these maps. Beth Israel is around tracts where both poverty and the non-White population are relatively low, and the population is already healthier. These areas have experienced the benefits of redlining and other forms of systemic exclusion, which also extend into healthcare infrastructure (Benjamins et al., 2021). BMC, however, is embedded within contiguous high-need tracts. This pattern and the strain on its resources raises important questions about equity in healthcare resource distribution and the sustainability of its service capacity. These findings align with the city’s population health agenda which prioritizes these neighborhoods along with East Boston and Jamaica Plain to close the life expectancy gap across neighborhoods (Boston Public Health Commission, 2025).

The operational and financial differences between St. Elizabeth and Carney are both tied to their geographies and eventual fates. The closure of Carney Hospital serves as a case study within the broader context of the United States’ expensive, market-driven healthcare system (Birn and Hellander, 2016). Within this system, a historically unprofitable hospital like Carney, serving a high-need community, could not find a viable bidder. In 2022, the total deficit of St. Elizabeth was $2.6 M relative to $445.7 M in revenue, whereas Carney reported a $22.6 M deficit on $98.1 M in revenue. Both hospitals had a high public payer mix, with 67.7 and 75.3% of gross patient service revenue derived from public payers, respectively (Center for Health Information and Analysis, 2024). Carney’s former discharge footprint in Dorchester/Mattapan aligns with the HH corridor shown in Figures 1–3. Yet, Carney could not find a viable bidder after the closure of Steward Health Care, despite accounting for 10% of all Mattapan and 12% of all Dorchester discharges (Center for Health Information and Analysis, 2024). Because Carney Hospital had relatively fewer beds, its closure produces only modest changes in tract-level accessibility (see Supplementary Figure 1).

The market failure is visually grounded in the spatial outliers identified in Figures 2, 3. These outliers offer especially valuable starting points for studying spillover effects of health inequity, as they identify areas where health outcomes diverge from the clustering of neighborhood demographics. Although prior work has focused on intra-hospital resource allocation (Ordu et al., 2021; Blake and Carter, 2002), the spatial mismatch in need and capacity documented here points to a critical gap at the inter-hospital level. Addressing this gap will require more formalized collaboration that enables hospitals in lower-need areas to directly support those serving high-need populations.

## 6 Conclusion

The present study has several limitations. First, the depth of the hospital-side analysis is limited by the lack of access to proprietary AHA Annual Survey data, which was cost-prohibitive for this study but would have provided more detailed information on admissions and discharges by payer type. The only publicly available hospital data in Boston comes from Center for Health Information and Analysis (2024) and includes aggregated statistics such as discharges to certain neighborhoods and bed counts that are used in this study. More comprehensive CHIA datasets and hospital-level detail are available primarily through restricted-use files that future studies should take advantage of to better understand micro spatial inequalities in Boston. Second, each household has more factors influencing their choice of healthcare provider. Even though the accessibility maps in the supplemental material represent potential spatial access, it still assumes that all residents within the 15-min catchment have equal access to hospital beds and that locations beyond the threshold are effectively inaccessible (Luo, 2004; Delamater, 2013). Thus, these results should not be interpreted as observed utilization.

Finally, the application of bivariate Moran’s *I* in the maps introduces another methodological constraint. Unlike univariate Moran’s *I*, the bivariate version does not inherently account for the correlation between the two variables at each spatial unit which complicates the interpretation of local spatial associations (Anselin, 2024). The bivariate Moran’s *I* assesses the degree of spatial autocorrelation between two distinct variables, but it does so without explicitly adjusting for the inherent correlation between these variables at each individual location. This can lead to potential confounding effects that complicate the interpretation of the strength and significance of spatial relationships. In this study, default permutation-based *p*-values (*p* < 0.05) were used without multiple-testing correction. Some local clusters may represent false positives due to multiple comparisons; thus, the findings should be interpreted carefully.

Regardless, methodologically, this study contributes to the literature by analyzing BiLISA maps in a local context alongside a 2-step floating catchment area (2SFCA) framework. This approach identifies smaller pockets of vulnerability, specifically High-Low (HL) and Low-High (LH) spatial outliers, that are obscured when health disparities are discussed only at the broad neighborhood level. In the specific context of Boston, these methods confirm established patterns of segregation and identifies tracts where health outcomes deviate from what poverty or racial composition alone would predict. Federal cuts to programs designed to support and protect our most vulnerable will place hospitals at risk of closure. Reductions in Medicare and Medicaid funding, for example, negatively affect all hospitals, but some are disproportionately impacted. Not every institution has the breathing room to withstand these losses. These spatial patterns are consistent with broader scholarship on structural racism in housing, financing, and healthcare systems (Mehrtash, 2025; Bailey et al., 2017). While this study does not test causal mechanisms, the clustering observed here aligns with decades of policy-driven spatial inequality. To make progress we must start reimagining health as a public good. This starts with lifting the weight from safety-net hospitals that collapse under community need, and it starts with reimagining who can get care and when. Future research should investigate these spatial patterns across different intersections using participatory, action-based, and community-centered methods specifically within Boston. More specifically, comparative studies across neighborhoods could show which policies and interventions most effectively counteract this legacy of structural racism in healthcare delivery.

## Data Availability

Publicly available datasets were analyzed in this study. This data can be found at: https://www.cdc.gov/places/; https://www.chiamass.gov/massachusetts-hospitals/; https://www.census.gov/programs-surveys/acs/.
