# Supplementary material for: Hospitals alike, hospitals different: spatial clustering of inequality around Boston’s hospitals
Source: Front Sociol. 2026 Jun 10;11:1736351. doi: 10.3389/fsoc.2026.1736351 (PMC13290557; doi:10.3389/fsoc.2026.1736351)
Supplement: Supplementary file 1 [file Table_1.DOCX]

Supplementary Online Materials

| Table 1: Factor loadings PCA results for Poor Health Index | | |
| --- | --- | --- |
|  | Factor Loadings | |
|  | 2017 | 2022 |
| Cancer | 0.89 | 0.89 |
| Coronary heart disease | 0.86 | 0.86 |
| Diagnosed diabetes | 0.81 | 0.82 |
| High blood pressure | 0.84 | 0.86 |
| High cholesterol | 0.8 | 0.81 |
| Stroke | 0.87 | 0.87 |


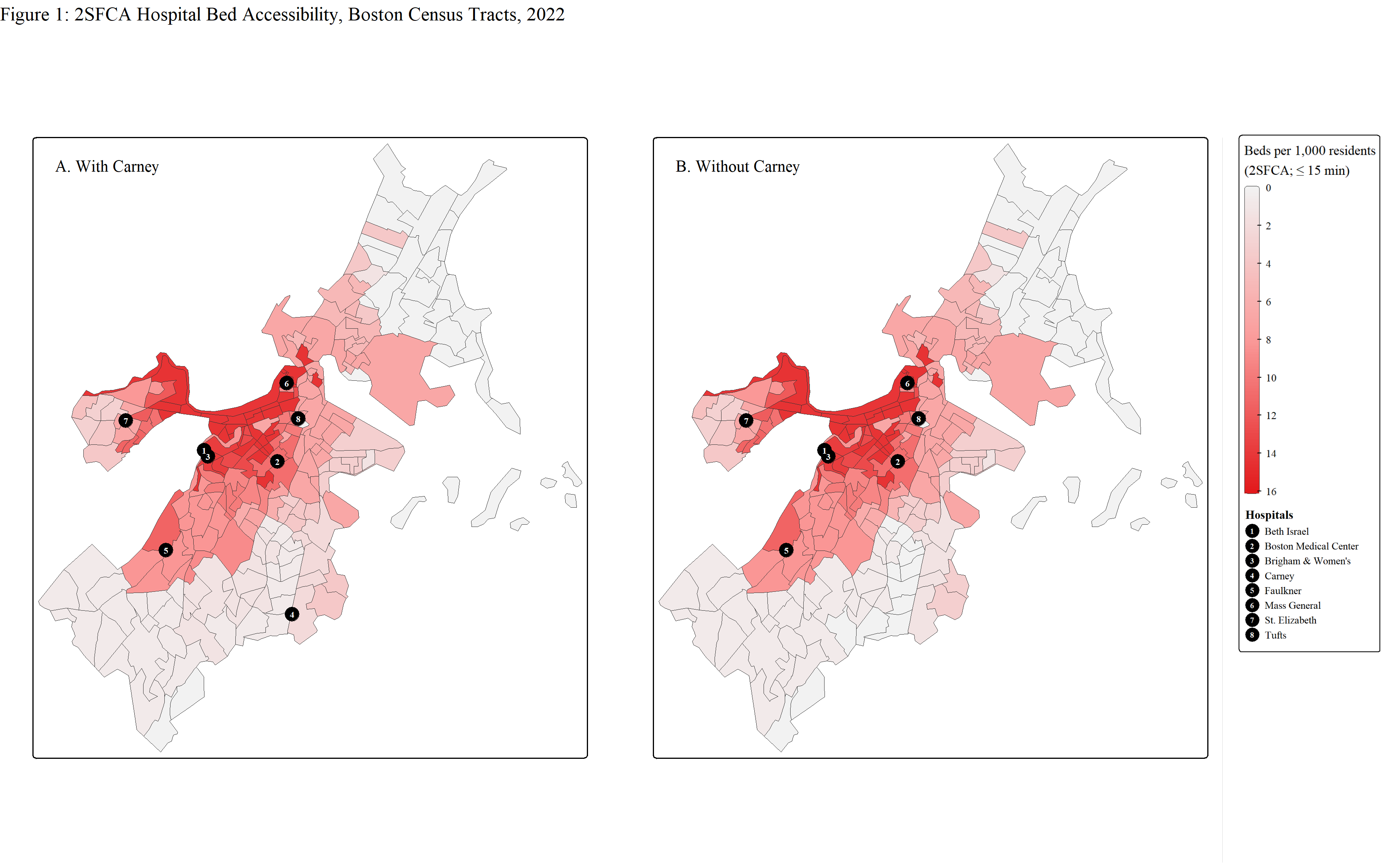


| **Table 2: Select Descriptive Statistics for Acute Care Hospitals in Boston** | | | | | | |
| --- | --- | --- | --- | --- | --- | --- |
|  | Emergency Department Visits | Total Gross Patient Service Revenue (M$) | Available Beds | Inpatient Discharges | Public Payer Mix^1^ | Inclusivity Ranking by Lown Institute^2^ |
| Brigham and Women's | 56537 | 8709.3 | 909 | 46511 | 55.80% | 2238 |
| Mass General | 109570 | 11820.7 | 1064 | 48426 | 60.30% | 882 |
| Beth Israel | 50520 | 3829.6 | 807 | 35996 | 57.70% | 1883 |
| Tufts | 37753 | 2410.4 | 428 | 16557 | 62.70% | 149 |
| Boston Medical Center | 107703 | 3908.1 | 461 | 21560 | 74.40% | 2 |
| St. Elizabeth | 25549 | 800.9 | 325 | 12697 | 67.70% | 312 |
| Faulkner | 28599 | 1018 | 171 | 7693 | 61.40% | 1774 |

1: Center for Health Information and Analysis. Massachusetts Hospital Profiles [Internet]. 2024. Available from: https://www.chiamass.gov/massachusetts-hospitals
2: Lown Institute. Hospitals Index for Social Responsibility [Internet]. 2024. Available from: https://lownhospitalsindex.org/rankings/?type=HospTyp_ACH

| Table 3: Changes in BiLISA maps using alternative spatial weighting | | |
| --- | --- | --- |
| Pairs | Weights | Agreement % |
| Non-white Population % and Poor Health Index | Inverse Distance Weights (IDW), p=1 | 84.6 |
| Non-white Population % and Poor Health Index | k-nearest neighbors, kNN(8) | 83.3 |
| Non-white Population % and Poor Health Index | Rook contiguity (1st order) | 93.2 |
| Non-white Population % and Poverty % | Inverse Distance Weights (IDW), p=1 | 87.6 |
| Non-white Population % and Poverty % | k-nearest neighbors, kNN(8) | 86.3 |
| Non-white Population % and Poverty % | Rook contiguity (1st order) | 92.7 |
| Poverty % and Poor Health Index | Inverse Distance Weights (IDW), p=1 | 85.5 |
| Poverty % and Poor Health Index | k-nearest neighbors, kNN(8) | 82.5 |
| Poverty % and Poor Health Index | Rook contiguity (1st order) | 93.2 |
